# Comparative analysis of gut microbiota in children with obstructive sleep apnea: assessing the efficacy of 16S rRNA gene sequencing in metabolic function prediction based on weight status

**DOI:** 10.3389/fendo.2024.1344152

**Published:** 2024-06-14

**Authors:** Hai-Hua Chuang, Chung-Guei Huang, Shih-Hsuan Chou, Hsueh-Yu Li, Chin-Chia Lee, Li-Ang Lee

**Affiliations:** ^1^ Department of Family Medicine, Chang Gung Memorial Hospital, Taipei Branch and Linkou Main Branch, Taoyuan, Taiwan; ^2^ School of Medicine, College of Medicine, Chang Gung University, Taoyuan, Taiwan; ^3^ Sleep Center, Metabolism and Obesity Institute, Linkou Chang Gung Memorial Hospital, Taoyuan, Taiwan; ^4^ School of Medicine, College of Life Science and Medicine, National Tsing Hua University, Hsinchu, Taiwan; ^5^ Department of Industrial Engineering and Management, National Taipei University of Technology, Taipei, Taiwan; ^6^ Department of Laboratory Medicine, Linkou Chang Gung Memorial Hospital, Taoyuan, Taiwan; ^7^ Department of Medical Biotechnology and Laboratory Science, Chang Gung University, Taoyuan, Taiwan; ^8^ Graduate Institute of Biomedical and Pharmaceutical Science, Fu Jen Catholic University, New Taipei City, Taiwan; ^9^ Biotools Co., Ltd., New Taipei City, Taiwan; ^10^ Department of Otorhinolaryngology - Head and Neck Surgery, Linkou Chang Gung Memorial Hospital, Taoyuan, Taiwan; ^11^ Taipei Wego Private Bilingual Senior High School, Taipei, Taiwan

**Keywords:** gut microbiome, synthetic 16S rRNA gene, read lengths, analysis pipelines, *Firmicutes*/*Bacteroidetes* ratio, obesity, obstructive sleep apnea

## Abstract

**Background:**

Analyzing bacterial microbiomes consistently using next-generation sequencing (NGS) is challenging due to the diversity of synthetic platforms for 16S rRNA genes and their analytical pipelines. This study compares the efficacy of full-length (V1–V9 hypervariable regions) and partial-length (V3–V4 hypervariable regions) sequencing of synthetic 16S rRNA genes from human gut microbiomes, with a focus on childhood obesity.

**Methods:**

In this observational and comparative study, we explored the differences between these two sequencing methods in taxonomic categorization and weight status prediction among twelve children with obstructive sleep apnea.

**Results:**

The full-length NGS method by Pacbio^®^ identified 118 genera and 248 species in the V1–V9 regions, all with a 0% unclassified rate. In contrast, the partial-length NGS method by Illumina^®^ detected 142 genera (with a 39% unclassified rate) and 6 species (with a 99% unclassified rate) in the V3–V4 regions. These approaches showed marked differences in gut microbiome composition and functional predictions. The full-length method distinguished between obese and non-obese children using the *Firmicutes*/*Bacteroidetes* ratio, a known obesity marker (*p* = 0.046), whereas the partial-length method was less conclusive (*p* = 0.075). Additionally, out of 73 metabolic pathways identified through full-length sequencing, 35 (48%) were associated with level 1 metabolism, compared to 28 of 61 pathways (46%) identified through the partial-length method. The full-length NGS also highlighted complex associations between body mass index z-score, three bacterial species (*Bacteroides ovatus*, *Bifidobacterium pseudocatenulatum*, and *Streptococcus parasanguinis* ATCC 15912), and 17 metabolic pathways. Both sequencing techniques revealed relationships between gut microbiota composition and OSA-related parameters, with full-length sequencing offering more comprehensive insights into associated metabolic pathways than the V3–V4 technique.

**Conclusion:**

These findings highlight disparities in NGS-based assessments, emphasizing the value of full-length NGS with amplicon sequence variant analysis for clinical gut microbiome research. They underscore the importance of considering methodological differences in future meta-analyses.

## 1 Introduction

The gut microbiota, primarily composed of *Firmicutes* and *Bacteroidetes* bacteria, plays a pivotal role in maintaining health and metabolic processes (1, 2). Existing evidence suggests that the gut microbiome holds a central role in regulating organismal energy balance (3), which includes the involvement of intestinal microbiota composition in intestinal plasticity and metabolism to sustain energy equilibrium (4). The gut microbiota of the human body is influenced by multiple exogenous and endogenous factors, such as genetic disposition, sex, age, diet (5), physical activity (6), sleep, pollutants (7), and others, leading to substantial variability across individuals and populations. An imbalance or disruption in the natural microbiota composition, referred to as dysbiosis, has been linked to a range of health conditions (8), including gastrointestinal disorders, autoimmune diseases, and metabolic conditions, such as obesity (9).

The *Firmicutes*/*Bacteroidetes* (F/B) ratio has been proposed as an obesity marker by some studies (10–12). Increased *Firmicutes* abundance and the F/B ratio in subjects with obesity are associated with disrupted energy metabolism (13). Reductions in *Bacteroides* and *Lactobacillus* induce lipid synthesis and storage in individuals with obesity via decreased bile acid concentrations (14). The altered gut microbiota and related metabolites contribute to weight gain by modulating central appetite and feeding behavior (15). In children, an increased presence of the *Firmicutes* phylum and a decreased presence of the *Bacteroidetes* phylum have been associated with high body mass index (BMI) (10). However, the association of the F/B ratio with obesity is contentious due to inherent methodological biases in microbiome analyses (12).

The 16S ribosomal RNA (rRNA) gene, a hallmark of prokaryotic life, encodes the RNA component of the ribosome’s small subunit and is essential for protein synthesis from mRNA. The gene encompasses conserved and variable regions (V1–V9) (16), enabling the 16S rRNA sequencing technique to serve as a cornerstone for microbial taxonomy and phylogeny (16, 17), reliably classifying bacteria up to the genus level (18). However, the precision of next-generation sequencing (NGS) for microbiome analysis varies with the sequencing approach, database, and targeted gene regions (19). Partial sequencing frequently lacks specificity beyond the genus level due to the selection of variable regions and read length inconsistencies (20–22). Although combining several hypervariable regions (usually V3–V4) may enhance resolution (23, 24), it can also lead to taxonomic misclassifications (25).

Advances in third-generation NGS technologies, characterized by long-read capabilities, allow for the sequencing of the complete 16S rRNA gene, which enhances the resolution of taxonomic classification (26). At the species level, full-length 16S rRNA gene sequencing has been shown to provide higher resolution compared to the V3–V4 regions, with improvements noted in alpha diversity, the frequency of relative abundance, and accuracy of identification (27). However, this technique is not without its challenges, which include the handling of unique tag sequences and the accurate identification of low-abundance variants (26, 28, 29). These technical hurdles contribute to an ongoing discourse regarding the relative merits of partial versus full-length sequencing for microbial analysis. This discussion highlights the necessity for methodological comparative studies, aiming to ascertain the most reliable approaches for analyzing the gut microbiome.

Obstructive sleep apnea (OSA) is a prevalent sleep disorder in children, with obesity increasingly recognized as a major risk factor alongside adenotonsillar hypertrophy (30). Obesity significantly correlates with the apnea-hypopnea index (AHI) in school-aged children with OSA (31). Emerging evidence suggests a genetic link between gut microbiota and OSA development (32), warranting further research into the effects of different 16S rRNA gene sequencing platforms and analytical pipelines on understanding the gut microbiota’s role in obesity among children with OSA.

We hypothesized that the full-length (V1–V9 region) synthetic 16S rRNA gene sequencing method will surpass the partial (V3–V4 region) method in (1) identifying and classifying the human gut microbiome, and (2) distinguishing patients with overweight or obesity (OWO) from those with normal weight (NW) using microbiota markers associated with obesity. In a sample of pediatric OSA patients with varying weight statuses, this study aimed to: (1) compare the results between full-length (V1-V9 region) and partial (V3–V4 region) 16S rRNA gene sequencing methods in analyzing the gut microbiome, and (2) investigate the implications of both methods on the identification of obesity-associated microbiota markers.

## 2 Methods

### 2.1 Study design and participants

This was an observation and comparative study. The participants were prospectively enrolled in an investigation of tonsil and gut microbiomes at the Department of Otolaryngology, Chang Gung Memorial Hospital (Linkou Main Branch, Taoyuan, Taiwan) between March 2017 and January 2019 (33). The Institutional Review Board of Chang Gung Medical Foundation approved the study (Approval No.: 201507279A3). Both parents and participants aged 6 years or older provided written informed consent. Our research adhered to the revised Declaration of Helsinki (34) and complied with the STROBE guidelines (35).

Children aged 5-12 years, exhibiting an AHI of ≥ 5.0 events/hour or an AHI of ≥ 2.0 events/hour with at least one associated morbidity (e.g., elevated blood pressure, daytime sleepiness, growth retardation), were considered eligible (36). We excluded patients with craniofacial, neuromuscular, or chronic inflammatory disorders (37). Those with acute inflammation or conditions requiring antibiotic treatment were only eligible for stool sample collection after a minimum of two weeks following remission.

**Figure 1:**
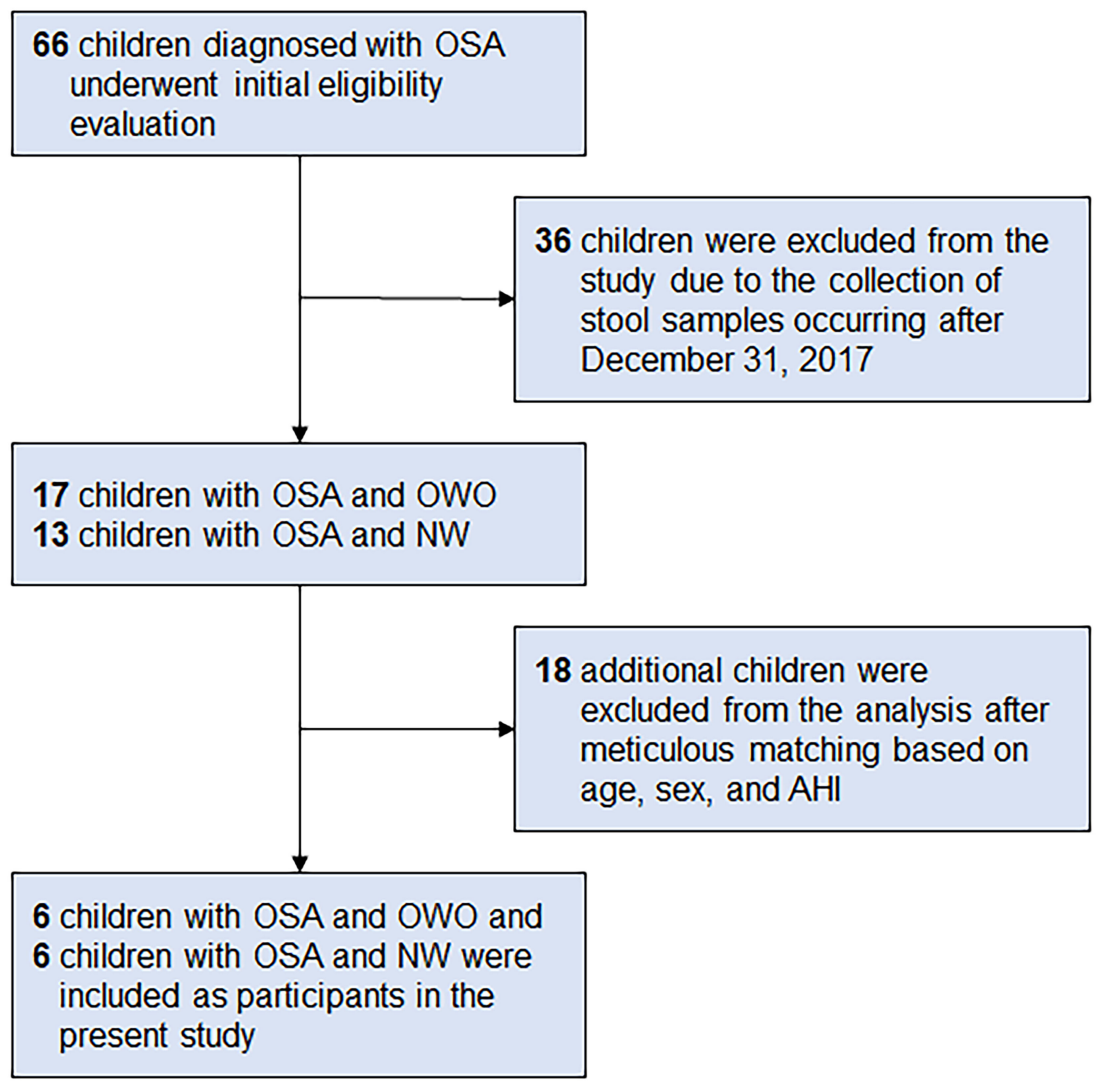
Participant flowchart. Following a rigorous matching process that considered age, sex, and apnea-hypopnea index (AHI), six children diagnosed with obstructive sleep apnea (OSA) and concurrent overweight/obesity (OWO) were meticulously selected. Correspondingly, an equivalent number of six children with OSA and normal weight (NW) were also included as participants in the present study. These participants were chosen from a total of 66 children who had been diagnosed with OSA.

From a database of 66 children (33), we meticulously selected six children with OWO and another six with NW based on age, sex, and AHI. The participants were subsequently categorized based on their BMI z-scores into the ‘OWO’ group (BMI z-score ≥ 1.0) and the ‘NW’ group (BMI z-score > -2.0 and < 1.0) (38). **Figure 1** displays the study flow diagram.

### 2.2 Polysomnography

Our study assessed various factors linked to OSA severity, including AHI, apnea index, arousal index, mean blood oxygen saturation (SaO_2_), minimum SaO_2_, total sleep time, and sleep stages. Following the 2012 American Academy of Sleep Medicine Manual guidelines (39), we employed standard full-night in-lab polysomnography. The detailed protocol for this polysomnography has been previously documented (37, 40, 41).

### 2.3 Stool sample collection and DNA preparation

Stool samples were collected, snap-frozen in liquid nitrogen, and stored at -80°C. DNA extraction utilized a fecal DNA isolation kit (MoBio Laboratories, USA). DNA concentrations were ascertained with a NanoPhotometer P360 system (Implen, USA) and standardized to 1 ng/ul for subsequent analyses of full-length or 5 ng/μl for subsequent analyses of partial length 16S rRNA amplicon sequencing.

### 2.4 Full-length 16S rRNA amplicon sequencing and taxonomy classification

The full-length 16S genes encompassing V1–V9 hypervariable regions were amplified using barcoded 16S gene-specific primers as detailed in PacBio^®^’s guide (42). Each primer is designed to contain a 5’ buffer sequence (GCATC) with a 5’ phosphate modification, a 16-base barcode and the degenerate 16S gene-specific forward or reverse primer sequences (Forward:5’Phos/GCATC- 16-base barcode - AGRGTTYGATYMTGGCTCAG -3’, Reverse: 5’Phos/GCATC- 16-base barcode – RGYTACCTTGTTACGACTT -3’) (43). The degenerate base identities are defined as follows: R = A or G; Y = C or T; M = A or C. A total of 2 ng of gDNA was used for the polymerase chain reaction (PCR), employing the KAPA HiFi HotStart ReadyMix (Roche, USA) under specified PCR conditions. Post-reaction, the PCR products were examined on a 1% agarose gel. Samples exhibiting a prominent band around 1,500 bp were selected and purified using AMPure PB Beads for subsequent library preparation.

The SMRTbell library preparation followed the PacBio^®^’s guidelines as above. Briefly, an equal molar amount of each barcoded PCR product was pooled, and between 500-1,000 ng of the pooled amplicon sample underwent DNA damage repair. This was followed by end-repair, A-tailing, and ligation steps to attach the universal hairpin adapters to double-stranded DNA region. After purification with AMPure PB beads to discard adapter dimers, the SMRTbell library was prepped with Sequel II primer 3.1 and Sequel II Binding Kit 3.1 for primer annealing and polymerase binding. Finally, sequencing was executed in the circular consensus sequence (CCS) mode on a PacBio^®^ Sequel IIe instrument to yield HiFi reads with a predicted accuracy (Phred Scale) of 30.

CCS reads were determined based on a minimum predicted accuracy of 0.9, with the least number of passes set at three, as per PacBio^®^’s official workflow using the SMRT Link software. Only CCS reads exceeding a quality score of Q30, termed Q30 HiFi reads, advanced to the succeeding phase. Post-demultiplexing, the HiFi reads underwent further processing using DADA2 (version 1.20; https://qiime2.org/) to extract amplicons with single-nucleotide resolution (44). Trimming and filtering were set to a maximum of two expected errors per read. The DADA2 algorithm can discern exact amplicon sequence variants (ASVs) from the full-length 16S rRNA gene with near-zero error rates. For each distinct sequence, tools such as the feature-classifier (45) and classify-consensus-vsearch (46) algorithm in QIIME 2 (v2022.11) were employed to annotate taxonomy classifications based on data from the National Center for Biotechnology Information (NCBI) 16S ribosomal RNA database (47). To evaluate sequence similarities among diverse ASVs, a multiple sequence alignment was executed using QIIME 2’s MAFFT (48) tool against the NCBI 16S ribosomal RNA database. A phylogenetic tree, illustrating the relationship of representative ASV sequences, was constructed using QIIME 2’s phylogeny FastTree (49).

### 2.5 Partial length 16S rRNA amplicon sequencing and taxonomy classification

We targeted the V3–V4 hypervariable region of the 16S rRNA gene for sequencing, amplified with specific primers: 341F (5’-CCTACGGGNGGCWGCAG-3’) and 806R (5’-GACTACHVGGGTAT CTAATCC -3’). Amplification followed the 16S Metagenomic Sequencing Library Preparation protocol (Illumina^®^, USA). PCR was carried out with 12.5 ng of genomic DNA, using KAPA HiFi HotStart ReadyMix (Roche, USA). The PCR products were verified via 1.5% agarose gel electrophoresis. DNA from samples with a prominent ~500 bp band was purified using AMPure XP beads (Beckman Coulter, USA). Quality assessment of the indexed PCR products was conducted using the Qubit 4.0 Fluorometer (Thermo Scientific, USA) and the Qsep100TM system (BiOptic, Taiwan). The sequencing library was constructed as per the aforementioned Illumina^®^ protocol, and sequencing was executed on an Illumina^®^ MiSeq platform, yielding paired 300-bp reads.

Post-sequencing, raw reads were demultiplexed based on barcodes. The paired-end reads underwent primer and adapter sequence removal via QIIME 2 cutadapt plugin (50). ASV construction involved the QIIME 2 DADA2 plugin (v2021.4), which facilitated quality filtering, dereplication, denoising, and more (44). Taxonomy classification and phylogenetic tree were performed as above.

### 2.6 Data analysis of gut microbiome

To account for sequence depth variations, ASVs were rarefied to the minimal sequence depth using QIIME’s script (single_rarefaction.py). Both alpha and beta diversity analyses utilized this normalized data. The community’s relative abundance and evenness accounting for alpha diversity were assessed using the Shannon, Simpson, and Peilou indices (51). For beta diversity, weighted and unweighted UniFrac were calculated (52). Statistical evaluations employed principal coordinate analysis (53) and non-metric multidimensional scaling (NMDS) (54). Furthermore, Welch’s t-test with the Benjamini-Hochberg procedure was performed to control false discovery rate using the STAMP software (v2.1.3) (55). The presence of statistically significant biomarkers was ascertained using the linear discriminant analysis effect size (LEfSe) analysis (56). Community structure differences were determined using analysis of similarity (57) and permutational multivariate analysis of variance (58). Functional abundances were predicted from full-length 16S rRNA data using Tax4Fun2 and the V3–V4 region for the Tax4Fun2 (59) and Kyoto Encyclopedia of Genes and Genomes (KEGG) orthology database (60). For highlighting pivotal functional profiles, the ggplot2 and microeco R packages were coupled with metastat statistics (61).

### 2.7 Statistical analysis

The normality of the continuous variables was assessed using the Kolmogorov-Smirnov test. For variables that followed a normal distribution, results were presented as the mean with standard deviation (SD). In cases where the distribution was non-normal, we reported the median alongside the interquartile range (IQR). To evaluate the differences between the two weight statuses, the unpaired Student *t*-test or Mann-Whitney *U* test were employed for continuous variables, while the Fisher exact test was used for categorical variables. For within-group comparisons, we applied either the paired Student t-test or the Wilcoxon signed-rank test, depending on the nature of the continuous variables. All statistical procedures were performed using multiple software tools: R (versions 4.3.1, R Foundation for Statistical Computing, Vienna, Austria), SPSS (version 27.0, IBM Corp., Armonk, NY, USA), and GraphPad Prism 10.0 for Windows (Graph Pad Software Inc., San Diego, CA, USA).

## 3 Results

### 3.1 Clinical characteristics of children with OSA and various weight status

Of the 66 children initially diagnosed with OSA, 36 were excluded because their stool samples were collected after December 31, 2017. Of the 30 children that remained, 17 had both OSA and OWO, and 13 had OSA with NW. Further exclusion based on age, sex, and weight status criteria left 12 participants for the final analysis: 6 children with concurrent OSA and OWO, and another 6 with OSA and NW, as illustrated in **Figure 1**. The final cohort consisted of 10 boys and 2 girls, with a median age of 6 years (IQR: 5–10 years). This is detailed in **Table 1**. The clinical variables were similar between the OWO and NW groups, except for the BMI z-score, which showed a significant difference (*p* = 0.002).

**Table 1:** Clinical characteristics of twelve pediatric patients with obstructive sleep apnea: a comparison between overweight/obesity and normal weight.

	Total	Overweight/obesity	Normal weight	*p*-Value
Age, years	6 (5–10)	8 (5–10)	6 (5–9)	0.589
Sex (girls/boys)	2/10	1/5	1/5	> 0.99
BMI z-score	0.8 (0.1–2.4)	2.4 (1.8–2.7)	0.1 (-0.8–0.4)	0.002
AHI, events/h	24.0 (20.0)	30.7 (27.0)	17.3 (6.4)	0.263
AI, events/h	4.0 (1.4–14.2)	5.9 (1.4–16.8)	4.0 (1.0–7.6)	0.699
Mean SaO_2_, %	97 (96–98)	97 (96–98)	98 (97–98)	0.699
Minimal SaO_2_, %	86 (82–91)	84 (79–91)	89 (83–91)	0.589
TST, minutes	336 (279–345)	340 (315–346)	311 (263–346)	0.394
N1 sleep, %	19 (10)	20 (12)	17 (9)	0.579
N2 sleep, %	36 (29–40)	37 (33–40)	30 (29–40)	0.589
N3 sleep, %	30 (7)	28 (6)	32 (7)	0.329
REM sleep, %	17 (7)	15 (7)	18 (8)	0.584

Data are displayed as mean (standard deviation), median (interquartile range), or number.

Between-group comparisons were evaluated using the unpaired Student t test or Mann-Whitney *U* test for continuous variables and the Fisher exact test for categorical variables.

AHI, apnea-hypopnea index; AI, apnea index; BMI, body mass index; REM, rapid eye movement; SaO_2_, blood oxygen saturation; TST, total sleep time.

### 3.2 Full-length 16S rRNA sequencing offers enhanced coverage but fewer ASVs

**Table 2** outlines the ASV counts, and corresponding sequence counts for each sample. With a Q score threshold of 30, the full-length 16S rRNA sequence via PacBio^®^ yielded significantly fewer ASVs than the V3–V4 region sequenced with Illumina^®^ (16044 (15554–18552) *vs.* 147952 (141946–152740), *p* < 0.001). Notably, almost all ASVs derived from full-length 16S rRNA sequencing were classified, resulting in a negligible rate of unassigned sequences (0–0.2% range) across phylum to species levels. In contrast, the V3–V4 region had a progressive increase in unassigned ASVs, especially from family (0.8%–14.5%) to species tiers (97.9%–99.9%). Moreover, the coverage rates from full-length V1–V9 region surpassed those of V3–V4 across all taxonomic levels (all *p* < 0.01).

**Table 2:** Microbial community analysis.

Group	Dataset	Sample	Read count	ASV count	Reads assigning taxonomic labels (coverage %)					
					Phylum	Class	Order	Family	Genus	Species
NW	Full-length	NW1	14995	12828	12828 (100)	12828 (100)	12828 (100)	12828 (100)	12828 (100)	12828 (100)
		NW2	14402	12090	12090 (100)	12090 (100)	12090 (100)	12090 (100)	12090 (100)	12090 (100)
		NW3	15859	13131	13131 (100)	13131 (100)	13131 (100)	13131 (100)	13131 (100)	13131 (100)
		NW4	18638	16271	16271 (100)	16271 (100)	16271 (100)	16271 (100)	16271 (100)	16271 (100)
		NW5	15325	12810	12810 (100)	12810 (100)	12810 (100)	12810 (100)	12810 (100)	12810 (100)
		NW6	18462	15895	15895 (100)	15895 (100)	15895 (100)	15895 (100)	15895 (100)	15895 (100)
	V3–V4	NW1	143507	100485	100460 (100)	100460 (100)	100460 (100)	99663 (99.2)	54567 (54.3)	119 (0.1)
		NW2	158072	104795	104769 (100)	104769 (100)	104608 (99.8)	103128 (98.4)	86779 (82.8)	355 (0.3)
		NW3	145091	91524	91294 (100)	91294 (99.7)	91294 (99.7)	90192 (98.5)	58282 (63.7)	55 (0.1)
		NW4	151220	98229	98207 (100)	98194 (100)	98180 (100)	97289 (99.0)	61759 (62.9)	666 (0.7)
		NW5	141425	106878	106863 (100)	106766 (99.9)	106663 (99.8)	104776 (98.0)	74092 (69.3)	491 (0.5)
		NW6	155020	113930	113930 (100)	113908 (100)	113584 (99.7)	112832 (99.0)	86653 (76.1)	1242 (1.1)
OWO	Full-length	OWO1	15728	14260	14233 (99.8)	14233 (99.8)	14233 (99.8)	14233 (99.8)	14233 (99.8)	14233 (99.8)
		OWO2	15496	13008	13008 (100)	13008 (100)	13008 (100)	13008 (100)	13008 (100)	13008 (100)
		OWO3	15798	13642	13642 (100)	13642 (100)	13642 (100)	13642 (100)	13642 (100)	13642 (100)
		OWO4	16228	14846	14846 (100)	14846 (100)	14846 (100)	14846 (100)	14846 (100)	14846 (100)
		OWO5	17416	15813	15813 (100)	15813 (100)	15813 (100)	15813 (100)	15813 (100)	15813 (100)
		OWO6	18582	16021	16021 (100)	16021 (100)	16021 (100)	16021 (100)	16021 (100)	16021 (100)
	V3–V4	OWO1	139392	93309	93130 (100)	93130 (99.8)	93130 (99.8)	91100 (97.6)	46526 (49.9)	1977 (2.1)
		OWO2	147824	108820	108812 (100)	108812 (100)	108759 (99.9)	107281 (98.6)	51333 (47.2)	266 (0.2)
		OWO3	135175	106320	106310 (100)	106310 (100)	106310 (100)	101350 (95.3)	71948 (67.7)	1139 (1.1)
		OWO4	148080	96100	96076 (100)	95987 (99.9)	95929 (99.8)	94367 (98.2)	46128 (48.0)	348 (0.4)
		OWO5	153246	98125	98125 (100)	97780 (99.6)	97000 (98.9)	83923 (85.5)	54190 (55.2)	365 (0.4)
		OWO6	150791	96096	96071 (100)	96046 (99.9)	95762 (99.7)	93752 (97.6)	43685 (45.5)	263 (0.3)

ASV, amplicon sequence variant; NW, normal weight; OWO, overweight/obesity.

### 3.3 Full-length 16S rRNA sequencing unveils greater bacterial species richness and elevated microbial diversities

**Figure 2:**
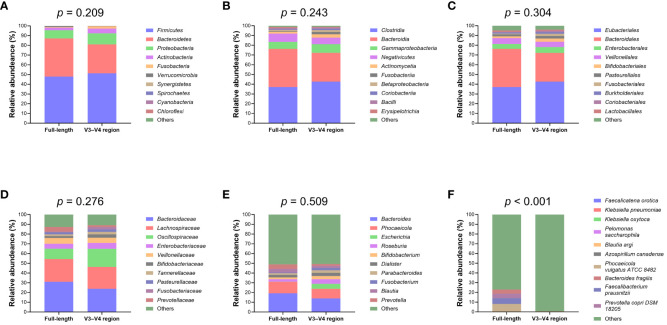
Comparative analysis of microbial taxonomic composition. Using the analysis of similarity test, taxonomic profiles showed no significant difference between the full-length and V3–V4 region of the 16S rRNA genes at the **(A)** phylum, **(B)** class, **(C)** order, **(D)** family, and **(E)** genus levels. Notably, **(F)** the species level exhibited marked variations between the full-length and V3–V4 region. Taxa that did not rank within the top ten most frequent and unassigned data are collectively termed “others”.

**Figure 3:**
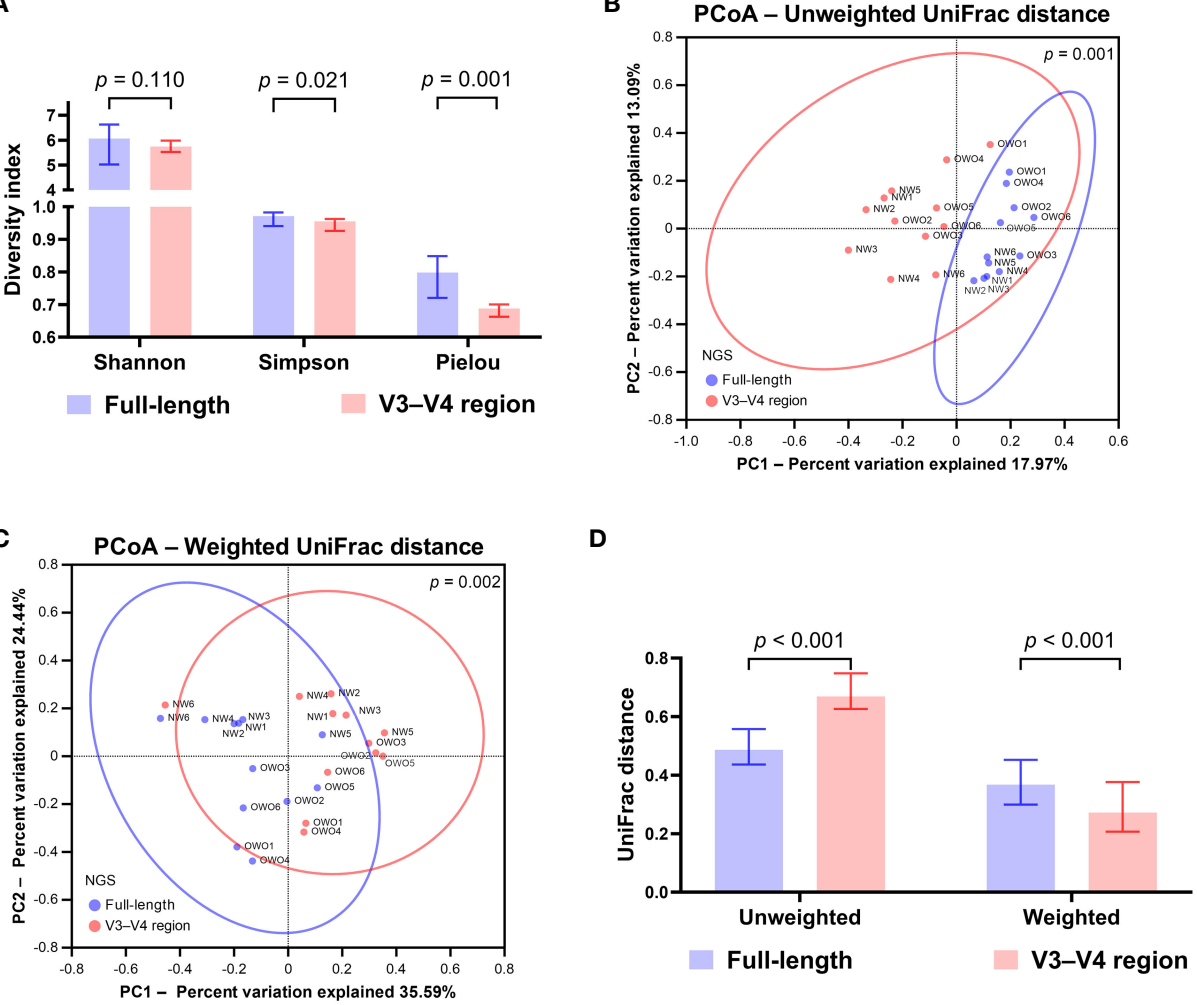
Differential analysis of alpha and beta diversity between sequencing methods. **(A)** The Wilcoxon signed-rank test revealed that the Shannon index did not differ significantly, while the Simpson and Pielou indexes were notably higher for the full-length method compared to the V3–V4 region. The principal coordinate analysis (PCoA) plots showcase the distributions of **(B)** unweighted and **(C)** weighted UniFrac distances for both sequencing methods. Their differences were found to be significant when analyzed with the permutational multivariate analysis of variance test. **(D)** Based on the Wilcoxon signed-rank test, the beta index for the full-length 16S rRNA sequencing was lower than the V3–V4 region using unweighted UniFrac distances. However, when weighted, the beta index was notably higher for the full-length method compared to the V3–V4 region. All data are presented as medians with interquartile ranges, and ellipses signify 95% confidence intervals.

Upon normalization by relative abundance, there were no significant disparities in the taxonomic profiles between full-length 16S rRNA sequencing and V3–V4 region from the phylum to the genus levels (**Figures 2A–E**; all *p* > 0.05). However, distinctions emerged at the species level (**Figure 2F**; *p* < 0.001). In terms of alpha diversity, the Simpson and Pielou indexes were both appreciably higher for full-length 16S rRNA sequencing as compared to the V3–V4 region (**Figure 3A**; *p* = 0.021 and *p* = 0.001, respectively). Delving further, it was observed that both unweighted (**Figure 3B**) and weighted (**Figure 3C**) UniFrac distances, as computed for individual samples from patients with NW and OWO, exhibited pronounced differences between the two NGS methodologies (*p* = 0.001 and *p* = 0.002, respectively). Regarding beta diversity (**Figure 3D**), the unweighted UniFrac distances associated with the full-length 16S rRNA sequencing were considerably lower than those of the V3–V4 region (*p* = 0.001). Conversely, the weighted UniFrac distances recorded for the full-length 16S rRNA sequencing surpassed those for the V3–V4 region (*p* = 0.001).

### 3.4 Full-length 16S rRNA sequencing offers a deeper insight into the disparities in gut microbiota between children with OWO and those with NW compared to the V3–V4 16S rRNA sequencing

**Figure 4:**
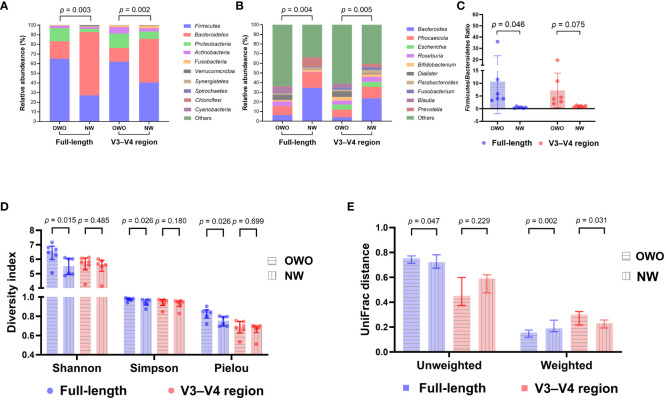
Comparative microbial composition in Overweight/Obese (OWO) and Normal Weight (NW) groups. **(A, B)** Using the analysis of similarity test, significant disparities in taxonomic profiles were found at both the phylum **(A)** and genus **(B)** levels when comparing the full-length and V3–V4 region of the 16S rRNA genes. **(C)** Specifically, the *Firmicutes*/*Bacteroidetes* ratio in the OWO group, as determined using the full-length methodology, was significantly elevated compared to the NW group (unpaired Student *t*-test). However, using the V3–V4 region methodology, the *Firmicutes/Bacteroidetes* ratios were similar between both groups. **(D, E)** Per the unpaired Mann-Whitney *U* test, the OWO group displayed markedly greater alpha **(D)** and beta **(E)** diversities than the NW group with the full-length method. However, using the V3–V4 region methodology, the majority of alpha and beta diversity metrics were analogous for both groups, save for the weighted UniFrac distance. Taxa not ranking within the top ten most frequent and all unassigned data are collectively labeled as “others”. Continuous data are depicted as either means ± standard deviations or as medians accompanied by interquartile ranges.

**Figure 5:**
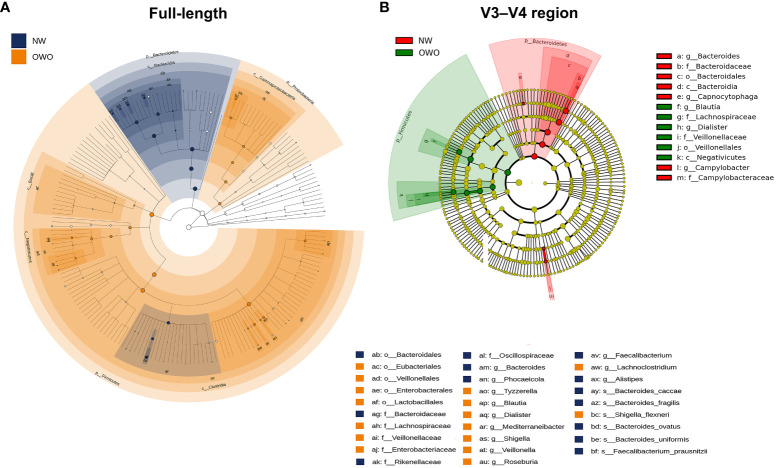
Cladograms comparing gut microbial composition in Overweight/Obesity (OWO) and Normal Weight (NW) groups. **(A)** The cladogram highlights 29 representative amplicon sequence variants having a linear discriminant analysis score ≥ 4, identified via the full-length method and LEfSe. **(B)** In contrast, the V3–V4 region methodology identified merely 13 representative amplicon sequence variants.

There were notable differences in ASVs at both the phylum and genus levels between the OWO and NW groups, as evidenced by both the full-length 16S rRNA sequencing and the V3–V4 region methodologies (**Figures 4A, B**; all *p* < 0.05). However, the full-length method provided deeper insights compared to the V3–V4 region. This distinction was apparent when evaluating the F/B ratio (**Figure 4C**), the alpha diversity (**Figure 4D**), beta diversity (**Figure 4E**), and the number of differentiated ASVs (**Figures 5A, B**), helping to better distinguish children with OWO from those with NW.

### 3.5 Both the full-length and V3–V4 16S rRNA sequencing methods reveal unique functional predictions of gut microbiota across weight categories

**Figure 6:**
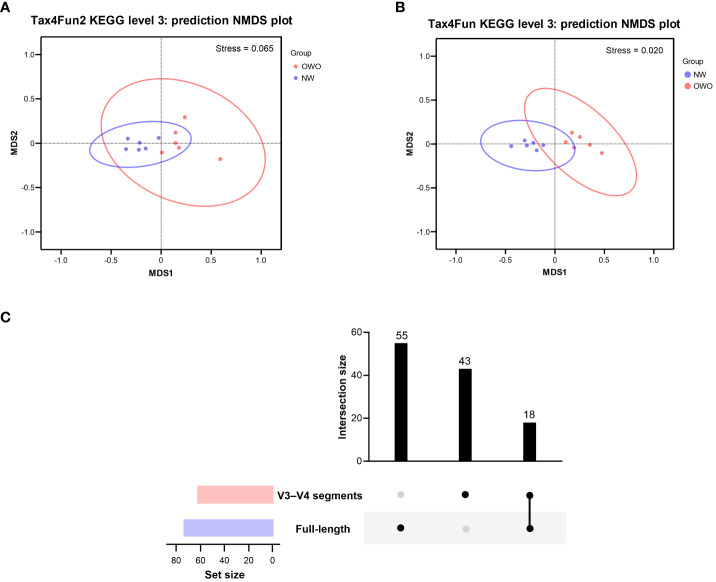
Functional profile analysis of gut microbiomes in children with Overweight/Obese (OWO) and Normal Weight (NW). **(A)** By integrating Tax4Fun2 with the NMDS test, we observed a distinct variation in the functional profiles of the two weight categories as per the KEGG orthology database. The stress values, which range from ≥ 0.05 to < 0.1, confirm the trustworthy representation of data after dimensionality reduction. **(B)** Using the older version, Tax4Fun, in conjunction with the NMDS test, the functional profile differences between the weight groups became evident once more. This reliable difference is accentuated by the strong representation in the visual plot with a stress value of < 0.05. **(C)** The UpSet plot showcases the overlap and unique pathways: 73 derived from the full-length 16S rRNA genes and another 61 from the V3–V4 segments. Notably, 18 predictive functions were consistently observed across both NGS methods.

Using the Tax4Fun2-based NMDS plot, a marked differentiation in the habitat-specific functional profile of gut microbiota emerged between children with OWO and those of NW status. This was clearly highlighted when analyzing data via the full-length 16S rRNA sequencing, paired with the level 3 pathway of the KEGG orthology database (stress value = 0.065) (**Figure 6A**). In parallel, the Tax4Fun NMDS plot, derived from V3–V4 segment data and aligned with the same functional prediction pathway, particularly pronounced divergences in functional profiles between the two weight groups (stress value = 0.021) (**Figure 6B**).

**Figure 7:**
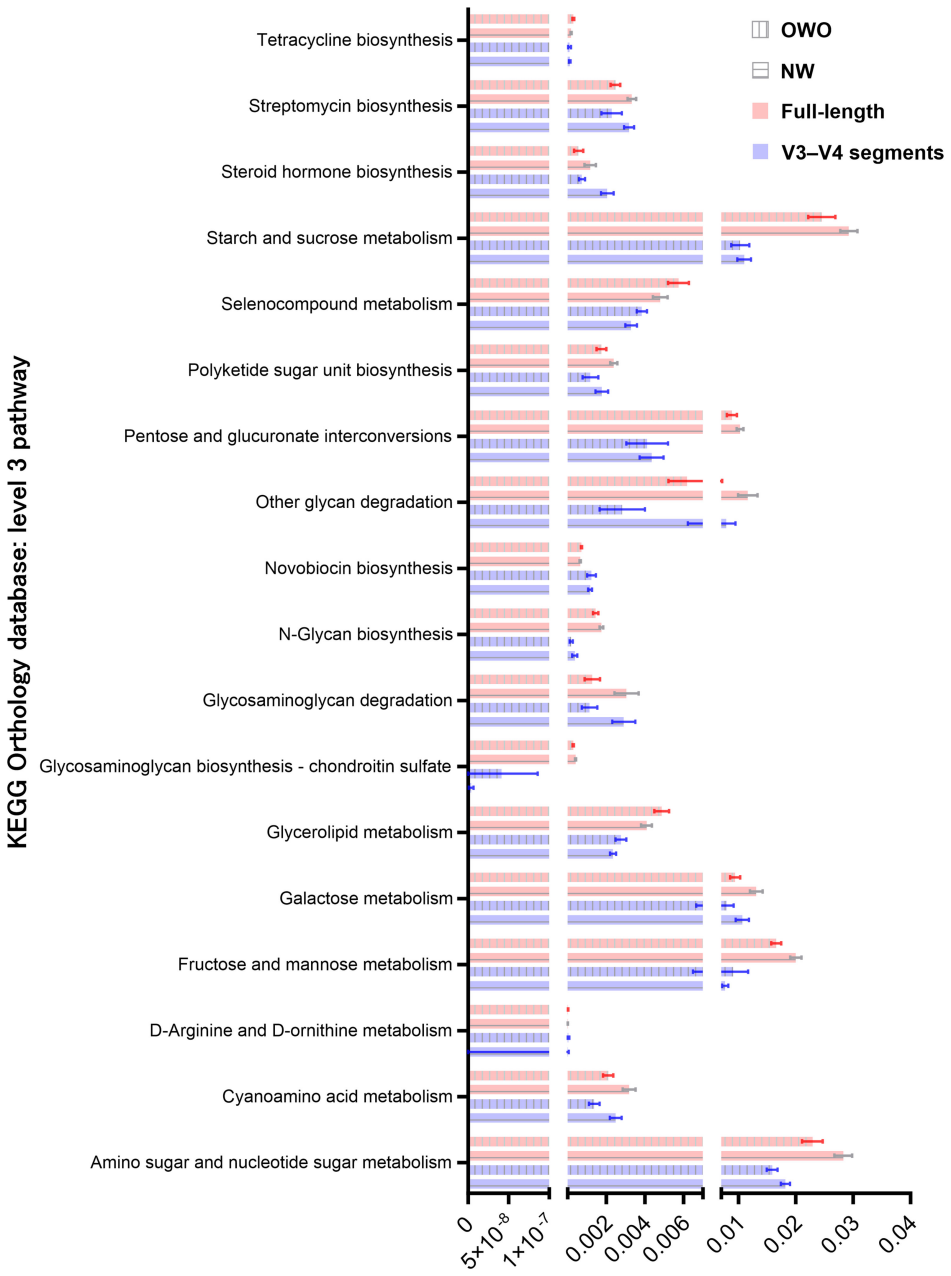
Comparison of predicted metabolic pathway abundance in gut microbiomes from children with Overweight/Obesity (OWO) and children with Overweight/Obesity (OWO) and Children with Normal Weight (NW). Through the columnar visualization, we get a clearer view of the pathway distributions, making it simpler to contrast and compare across the weight classifications and the two NGS technologies.

By harnessing the Welch’s *t*-test in tandem with the Benjamini-Hochberg procedure to manage the false discovery rate, we pinpointed 73 distinct level 3 pathways using full-length 16S rRNA genes and another 61 pathways using the V3–V4 region (**Figure 6C**). All pathways were identified with both *p*- and *q*-values falling below 0.05. Intriguingly, both NGS methods shared 18 predictive functions. Difference in the proportion of overlapping level 3 functional predictions were not significant (25% vs 30%, *p* = 0.562). **Figure 7** delves deeper into these shared 18 level 3 pathways. While the majority (*n* = 16) exhibited consistent distribution patterns across both methods, two pathways — specifically, fructose and mannose metabolism, and glycosaminoglycan biosynthesis-chondroitin sulfate — displayed distinct patterns between the two methods.

Additionally, of the 73 identified through full-length methodology, 35 (48%) were affiliated with the level 1 metabolism pathway, while from the 61 pathways discerned through the V3–V4 region, 28 (46%) resonated with the same category. Interestingly, five metabolism pathways—comprising fructose and mannose metabolism, galactose metabolism, tetracycline biosynthesis, steroid hormone biosynthesis, and pentose and glucuronate interconversions—exhibited consistency between the two methodologies. The proportion of overlapping level 1 metabolism pathways were similar (86% vs 82%, *p* = 0.737).

### 3.6 Association between weight-related gut bacterial species and metabolic pathways

**Figure 8:**
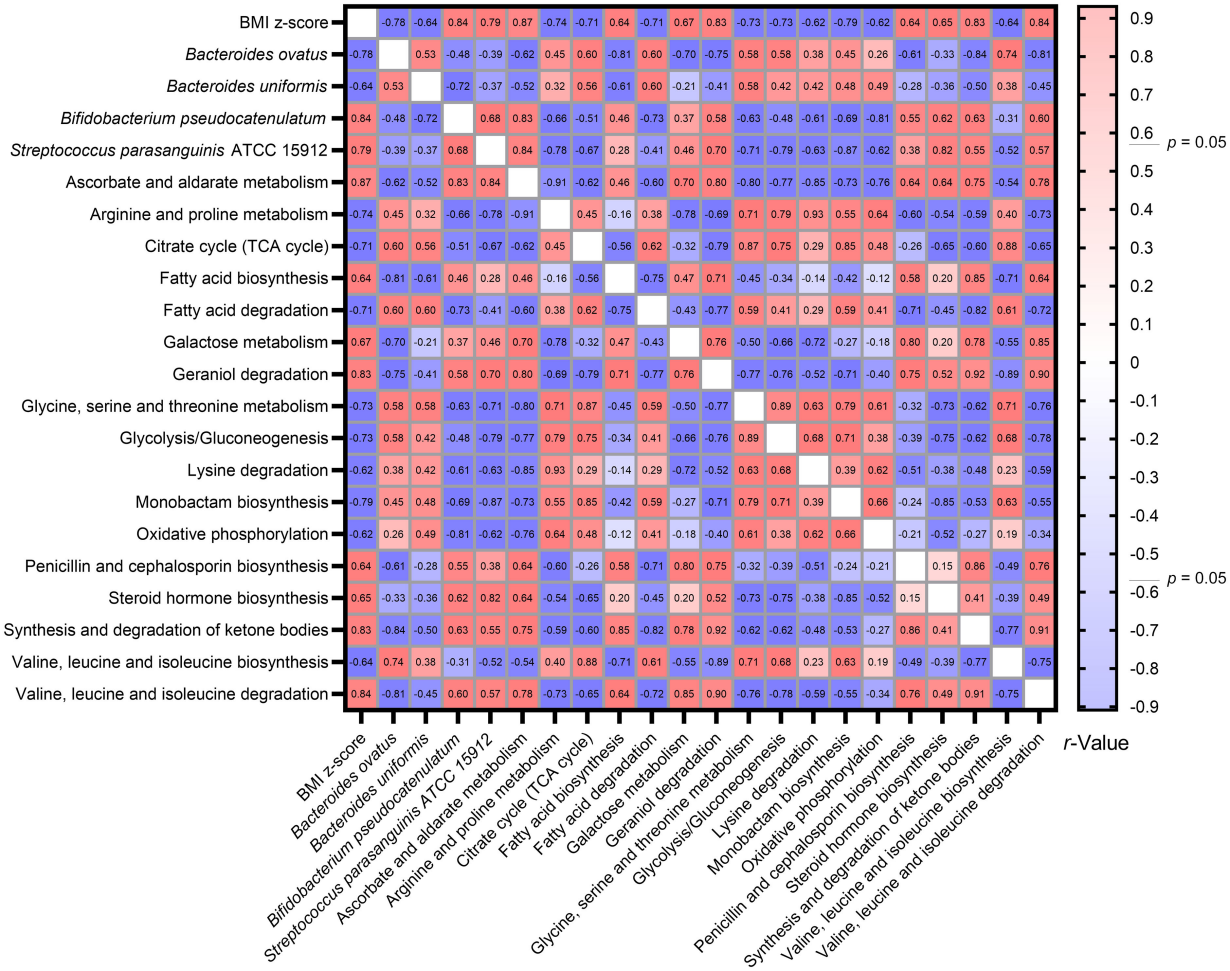
Heatmap illustrating the spearman correlations Between BMI Z-score, specific gut bacteria, and metabolic pathways. By consolidating findings from Welch’s *t*-test, LEfSe, and Tax4Fun2, we identified intricate associations between the BMI z-score, four gut bacterial species, and 17 metabolic pathways. The Spearman correlation test facilitated this observation.

Utilizing the Spearman correlation test, a set of bacteria—*Bacteroides ovatus*, *Bifidobacterium pseudocatenulatum*, and *Streptococcus parasanguinis* ATCC 15912—were identified in association with BMI z-score. These were discerned through the combined use of Welch’s *t*-test and the Benjamini-Hochberg procedure. Additionally, *Bacteroides uniformis* was singled out via the LEfSe method as having relevance to BMI z-score. Concurrently, an analysis using Tax4Fun2 highlighted 17 metabolic pathways that bore associations with the BMI z-score (**Figure 8**).

**Figure 9:**
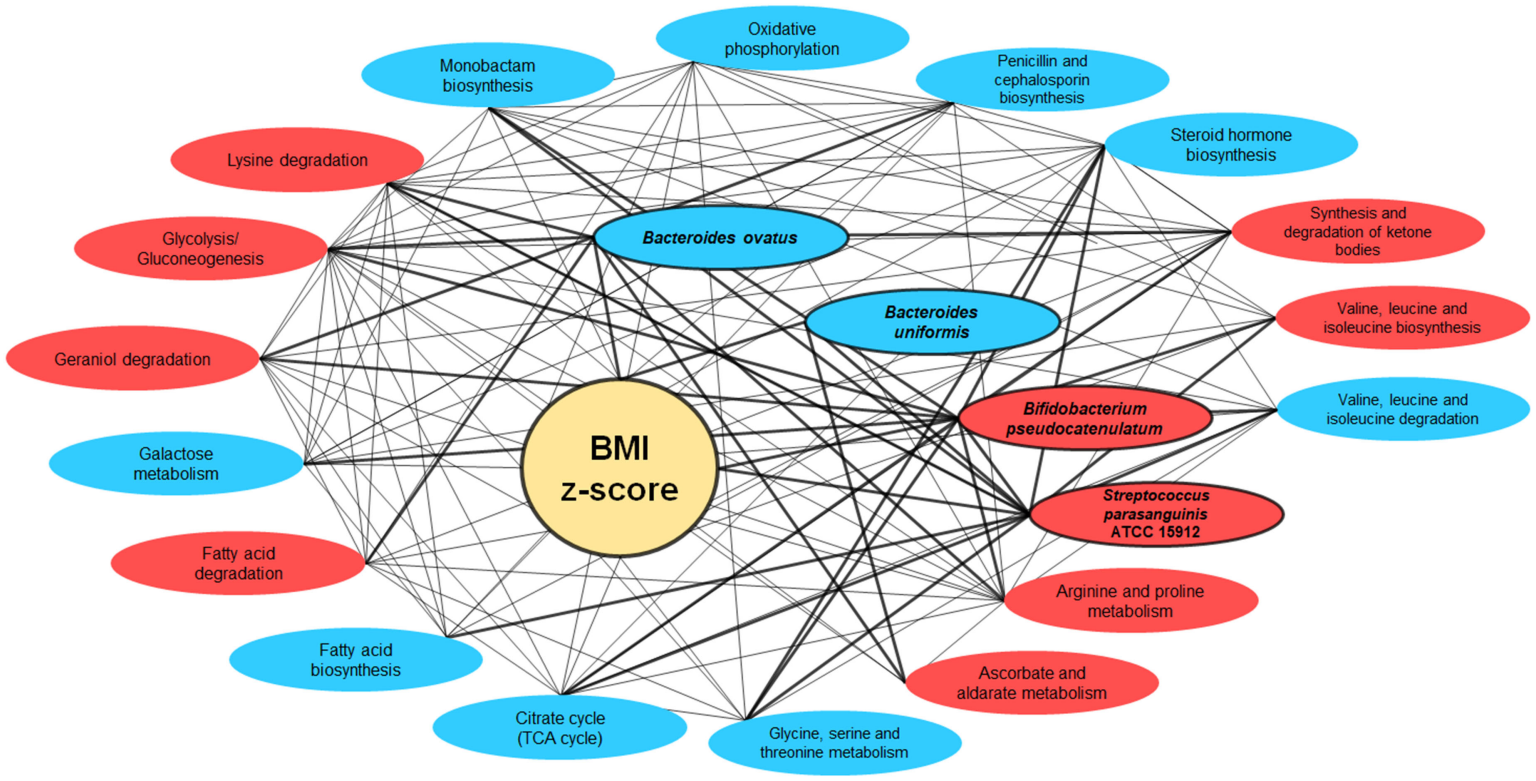
Network interactions illustrating the correlation between BMI Z-score, gut bacteria, and metabolic pathways. This network graph elucidates the complex relationships between BMI z-score, the gut bacteria *Bacteroides ovatus*, *Bifidobacterium pseudocatenulatum*, and *Streptococcus parasanguinis* ATCC 15912, and various metabolic pathways. Nodes represent either bacterial species or metabolic pathways, with red ellipses indicating associations with overweight/obesity and blue for normal weight. The interconnecting lines represent correlation strengths, with thicker lines denoting stronger associations (correlation coefficient of *r*-value ≥ 0.60).

The intricate interconnections amongst BMI z-score, the four gut bacterial species, and the metabolic pathways are vividly depicted in the network graph. This representation underscores the multifaceted associations between BMI z-score and these critical components of the gut microbiome. Strikingly, the trio of bacteria—*Bacteroides ovatus*, *Bifidobacterium pseudocatenulatum*, and *Streptococcus parasanguinis* ATCC 15912—showcased active interactions with the metabolic pathways (**Figure 9**).

### 3.7 Correlations between gut microbiota composition, functional predictions, and polysomnographic parameters

Comprehensive 16S rRNA gene sequencing revealed that alpha diversity indices were not correlated with polysomnographic measures. However, distinct genera demonstrated significant associations: *Bifidobacterium* levels correlated with AHI and *Escherichia* with AI. Additionally, *Phocaeicola* abundance was inversely related to AI, and *Bifidobacterium* abundance was inversely associated with minimal SaO_2_. Extended analysis using the V3–V4 regions of the 16S rRNA gene confirmed these relationships and further identified a significant correlation between *Bifidobacterium* and AI (**Table 3**).

**Table 3:** Spearman correlations of alpha diversity indices and top 10 gut microbiota genera with polysomnographic parameters in pediatric obstructive sleep apnea.

Predicted function	AHI	AI	Mean SaO_2_	Minimal SaO_2_
Full-length 16S rRNA sequencing				
Shannon	0.29	-0.04	-0.39	-0.43
Simpson	0.25	-0.14	-0.44	-0.41
Pielou	0.16	-0.15	-0.34	-0.35
*Bacteroides*	-0.09	0.07	0.10	0.01
*Phocaeicola*	-0.41	-0.67*	-0.14	0.36
*Roseburia*	0.05	-0.34	-0.43	-0.22
*Prevotella*	0.13	0.39	0.32	-0.23
*Escherichia*	0.38	0.84*	0.07	-0.24
*Blautia*	0.48	0.07	-0.49	-0.39
*Dialister*	-0.33	-0.31	0.09	0.36
*Veillonella*	0.09	0.30	0.34	0.07
*Bifidobacterium*	0.58*	0.56	-0.23	-0.69*
*Parabacteroides*	0.01	-0.02	-0.05	-0.17
V3–V4 16S rRNA sequencing				
Shannon	-0.15	-0.32	-0.31	0.03
Simpson	-0.18	-0.33	-0.27	-0.08
Pielou	-0.10	-0.31	-0.41	-0.07
*Bacteroides*	-0.04	0.18	0.10	-0.05
*Phocaeicola*	-0.48	-0.58*	0.07	0.54
*Escherichia*	0.12	0.71*	0.31	-0.05
*Roseburia*	0.13	-0.52	-0.55	-0.15
*Bifidobacterium*	0.61*	0.59*	-0.23	-0.66*
*Dialister*	-0.34	-0.32	0.08	0.32
*Parabacteroides*	-0.08	0.06	0.18	0.03
*Fusobacterium*	0.19	0.13	-0.17	-0.04
*Blautia*	0.17	0.22	-0.06	-0.28
*Prevotella*	0.06	0.08	0.01	0.21

Spearman’s *r*-values are used to represent correlations. Significant correlations are marked as follows: **p < 0.01, *p < 0.05. AHI, apnea-hypopnea index; AI, apnea index; SaO_2_, blood oxygen saturation.

Bold font indicates statistically significant differences (*p* < 0.05).

Metabolic function predictions based on full-length 16S rRNA gene sequencing indicated significant associations between microbial metabolism in diverse environments, propanoate metabolism, and AI, as well as between glycine, serine, and threonine metabolism and mean SaO_2_. Conversely, sequencing of the V3–V4 regions elucidated a notable association of peptidoglycan biosynthesis with minimal SaO_2_ (**Table 4**).

**Table 4:** Spearman correlations between top 20 predicted level 3 metabolic functions of gut microbiota and polysomnographic parameters in pediatric obstructive sleep apnea.

Predicted function	AHI	AI	Mean SaO_2_	Minimal SaO_2_
Full-length 16S rRNA sequencing				
Biosynthesis of secondary metabolites	-0.13	0.11	0.12	-0.23
Biosynthesis of antibiotics	0.35	0.34	-0.28	-0.53
Microbial metabolism in diverse environments	0.52	0.72**	-0.09	-0.32
Biosynthesis of amino acids	-0.11	-0.12	0.10	-0.25
Carbon metabolism	-0.28	0.10	0.39	0.34
Amino sugar and nucleotide sugar metabolism	-0.26	-0.39	0.13	0.45
Purine metabolism	0.24	0.46	0.17	-0.11
Pyrimidine metabolism	0.09	0.10	0.17	-0.23
Pyruvate metabolism	-0.11	0.24	0.18	0.12
Glycolysis/Gluconeogenesis	0.43	0.15	-0.40	-0.37
Starch and sucrose metabolism	0.00	-0.33	-0.16	-0.12
Galactose metabolism	-0.17	-0.36	-0.03	0.27
Glyoxylate and dicarboxylate metabolism	0.08	0.17	-0.07	0.13
Fructose and mannose metabolism	0.49	0.34	-0.34	-0.03
Carbon fixation pathways in prokaryotes	-0.49	-0.08	0.52	0.43
Cysteine and methionine metabolism	0.00	0.32	0.33	-0.27
Propanoate metabolism	0.43	0.58*	-0.17	-0.09
Alanine, aspartate and glutamate metabolism	-0.17	-0.50	-0.17	0.34
Glycine, serine and threonine metabolism	-0.41	0.15	0.59*	0.24
V3–V4 16S rRNA sequencing				
ABC transporters	0.22	-0.09	-0.28	-0.12
Two-component system	-0.08	-0.04	0.03	0.27
Purine metabolism	0.39	0.57	-0.27	-0.53
Pyrimidine metabolism	0.13	-0.21	-0.08	-0.18
Aminoacyl-tRNA biosynthesis	0.32	0.36	-0.22	-0.46
Starch and sucrose metabolism	0.08	0.08	-0.04	-0.12
Amino sugar and nucleotide sugar metabolism	0.10	0.21	-0.03	-0.11
Arginine and proline metabolism	-0.14	-0.42	0.17	0.15
Ribosome	0.06	0.13	-0.05	-0.38
Fructose and mannose metabolism	-0.09	0.10	0.11	-0.04
Alanine, aspartate and glutamate metabolism	-0.04	-0.40	0.03	0.03
Porphyrin and chlorophyll metabolism	0.16	0.18	-0.13	-0.04
Homologous recombination	0.02	-0.14	0.09	-0.05
Oxidative phosphorylation	-0.08	-0.34	0.12	0.05
Peptidoglycan biosynthesis	0.47	0.51	-0.46	-0.58*
Cell cycle - Caulobacter	0.29	-0.20	-0.31	-0.25
Nitrogen metabolism	-0.40	0.11	0.46	0.41
Methane metabolism	0.36	-0.13	-0.44	-0.46
RNA degradation	-0.15	0.28	0.21	0.06

Spearman’s *r*-values are used to represent correlations. Significant correlations are marked as follows: **p < 0.01, *p < 0.05. AHI, apnea-hypopnea index; AI, apnea index; SaO_2_, blood oxygen saturation.

Bold font indicates statistically significant differences (*p* < 0.05).

## 4 Discussion

This study investigated the gut microbiome of twelve pediatric OSA patients, half with OWO and half without, using both the V3-V4 region and full-length 16S rRNA sequencing methods. The clinical characteristics were comparable the two groups except for the BMI z-score. The full-length sequencing, despite producing fewer ASVs, outperformed the V3–V4 region in terms of taxonomic coverage. It also resulted in fewer unassigned sequences and exhibited significant variations in both alpha and beta diversities. Interestingly, the full-length sequencing provided deeper insights into the differences in gut microbiota composition between children with OWO and children with NW compared to the V3–V4 approach. Functionally, each method highlighted unique metabolic pathways associated with gut microbiota across the different weight categories. The full-length sequencing was particularly effective in identifying bacterial species linked to BMI z-score, emphasizing their potential significance in the dynamics of weight-related gut microbiome. **Table 5** provides a comprehensive comparison of these two NGS methods, including microbiota bioinformatics, correlation with weight status, and functional predictions of microbial communities in stool samples. Subsequent sections will delve deeper into the detailed findings.

**Table 5:** Comparison of full-length and V3–V4 region 16S rRNA gene sequencing methods in assessing gut microbiome across different weight groups in children.

Items/Parameters	Full-length	V3–V4 region
NGS methodologies		
NGS technology	Third-generation	Second-generation
Current application	Emerging	Preferred method
PCR primers	V1–V9 region	V3–V4 region
NGS platform	Pacbio^®^	Illumina^®^
Amplicon length	1500 bp	300 bp
Bioinformatics platform	QIIME 2 (version 2022.1)	QIIME 2 (version 2021.4)
Analysis pipelines	DADA2 (version 1.20)	DADA2 (version 2021.4)
Taxonomic database	NCBI (2020.7)	NCBI (version 2022.1)
Microbiota bioinformatics		
Read count	16044 (15554–18552)	147952 (141946–152740)
ASV count	13951 (12873–15875)	99357 (96097–106739)
Coverage rate	High	Low
Identified species	Numerous	Limited
Similarity of relative abundance	Similar (except at species level)
Alpha diversity	High	Low
Beta diversity (unweighted)	Low	High
Beta diversity (weighted)	High	Low
Correlation with weight status (small sample size)		
Taxonomic profile	Present	Present
*Firmicutes*/*Bacteroidetes* ratio	More distinct	Less distinct
Alpha diversity	Present	Absent
Beta diversity, unweighted	More distinct	Less distinct
Beta diversity, weighted	Present	Present
Representative ASVs	Higher	Lower
Functional prediction of gut microbiota		
Software	Tax4Fun2	Tax4Fun
Database	KEGG	KEGG
Differentiated level 3 pathway (overlapping/unique)	18/55	18/43
Differentiated metabolic pathway (overlapping/unique)	5/31	5/23
Note	Highlighted inconsistency in fructose and mannose metabolism representations	
Translational significance	Elucidating gut bacterial species can lead to more targeted mechanistic research, enriching our understanding of the complex gut interactions	Embracing a universally accepted method for gut microbiome study ensures consistent and comparable results, fostering collaboration and field advancements

Data are presented as median (interquartile range) or number.

ASV, amplicon sequence variant; KEGG, Kyoto Encyclopedia of Genes and Genomes; NCBI, National Center for Biotechnology Information; NGS, next-generation sequencing; PCR, polymerase chain reaction.

### 4.1 16S rRNA sequencing methods: full-length or V3–V4 region?

In this research, we focused on comparing two sequencing methods: full-length sequencing and the V3–V4 region sequencing of the 16S rRNA gene. One of the most striking observations was that full-length sequencing demonstrated superior taxonomic coverage compared to the V3–V4 region as previous studies (27, 62). This suggests that the full-length method captures a broader spectrum of microbial diversity, potentially providing a more comprehensive picture of the microbial community. This comprehensive taxonomic resolution could be particularly beneficial in studies where fine-scale differences in microbial communities are of interest (63).

While the full-length sequencing yielded fewer ASVs after denoising, it’s crucial to consider the quality and relevance of these ASVs. Fewer ASVs doesn’t necessarily imply reduced microbial diversity but may indicate reduced noise or spurious sequences (64). The reduced rate of unassigned sequences in the full-length method further underscores its precision. Unassigned sequences, often a source of ambiguity in microbial studies, can stem from sequencing errors, chimeric sequences, or genuine novel sequences that aren’t yet represented in reference databases (65).

Despite similar relative abundances of both methods, the significant variations in both alpha (within-sample diversity) and beta diversities (between-sample diversity) between the two methods have substantial implications. The higher alpha diversity in the full-length sequencing might suggest that it can detect a wider range of taxa within a single sample, potentially unearthing rare or low-abundance species that might be missed by the V3–V4 method (66). Differences in beta diversity, on the other hand, could imply that community compositions derived from the two methods might not be directly comparable (22). This is pivotal for studies looking at differences between groups, such as those based on health status, geography, or other variables.

While our results highlight the potential advantages of full-length 16S rRNA gene sequencing, researchers should carefully consider their objectives before selecting a method. For instance, studies that require rapid results or are constrained by budget might still benefit from the V3–V4 region due to its established protocols, faster turnaround, and lower per-case costs (67). Conversely, projects demanding in-depth taxonomic resolution might favor the full-length approach.

### 4.2 Potential applications in studying weight-related gut microbiome

The observed superiority of full-length sequencing in discerning the differences in gut microbiota composition and functional prediction between OWO and NW groups versus the V3–V4 approach is a significant finding. The enhanced depth offered by full-length sequencing might be analogous to viewing an image in higher resolution (66). Where the V3–V4 approach might provide a general view of the microbiota landscape, full-length sequencing offers a detailed map. In this study, the performance of the full-length sequencing to differentiate children with OWO from children with NW was better than that of the V3–V4 approach, regarding F/B ratio, alpha diversity, and beta diversity. Therefore, the full-length sequencing is particularly cost-effective when the sample size was relatively small. This can be especially significant when identifying minor bacterial taxa that may have substantial impacts on host physiology (67, 68). For example, the full-length method revealed six bacterial species of 29 representative ASVs, whereas the V3–V4 region methodology identified 0 bacterial species of 13 representative ASVs. Furthermore, *Bacteroides ovatus*, *Bacteroides uniformis*, *Bifidobacterium pseudocatenulatum*, and *Streptococcus parasanguinis* ATCC 15912 were closely related to BMI z-score by using the full-length method.

The differences in functional predictions identified by each sequencing method underscore the complex and multifaceted nature of the gut microbiome’s role in host metabolism (69). In this study, we pinpointed 73 distinct level 3 pathways using full-length 16S rRNA genes and another 61 pathways using the V3–V4 region. Differences in gut microbiota composition can impact nutrient absorption, gut barrier function, inflammation, and interactions with other organ systems (70). Furthermore, the metabolic pathways, whether involved in short-chain fatty acid production, amino acid metabolism, or other processes, might hold the key to understanding how gut microbiota influence weight and overall health (71–73). Using the full-length method, 35 differentiated metabolic pathways were identified, whereas the V3–V4 region method revealed 28 differentiated metabolic pathways. Furthermore, *Bacteroides ovatus*, *Bifidobacterium pseudocatenulatum*, and *Streptococcus parasanguinis* ATCC 15912 showcased active interactions with the metabolic pathways and closely related to BMI z-score by using the full-length method. Therefore, beyond just identifying which bacterial species are present, the full-length sequencing might offer insights into the potential functions of these bacteria. Moreover, understanding how various strains and species are related can give insights into how the gut microbiome might have evolved in response to dietary, environmental, or other external factors in children with OWO versus children with NW.

The ability of full-length sequencing to associate specific bacterial species with the BMI z-score is not just a technical achievement but also a clinically relevant finding. Such associations might provide the foundation for personalized therapeutic interventions. For instance, seaweed *Undaria pinnatifida* administration significantly increased lean-related *Bacteroides ovatus* and short-chain fatty acids and tricarboxylic acid cycle intermediates and decrease high-fat diet-induced body weight gain in high-fat diet-fed mice (74). As mentioned above, carbohydrate, amino acids, and lipid metabolisms are weight-related metabolic pathways. Interestingly, the role of *Bifidobacterium pseudocatenulatum* on these pathways is strain dependent. For example, *Bifidobacterium pseudocatenulatum* CECT 7765 can reduce both metabolic and immunological dysfunctions related to obesity in HFD-fed mice (75), whereas *Bifidobacterium pseudocatenulatum* JCLA3 involves the carbohydrate and amino acids metabolism (76). *Streptococcus parasanguinis* ATCC 15912 is an atypical viridans *streptococcus* (77) and its clinical significance is not well studied. In this study, *Streptococcus parasanguinis* ATCC 15912 is positively associated with BMI z-score and involves amino acid and lipid metabolic pathways. This is a novel finding; however, its exact mechanism of weight gaining should be further investigated. Nevertheless, understanding the metabolic pathways prevalent in different weight categories could guide dietary recommendations.

While the study focuses on weight differences, it indirectly sheds light on the broader interactions between the host and its microbiota. The gut microbiota’s influence isn’t limited to weight but extends to the immune system, mental health, and more (78, 79). The identified differences in gut microbiota composition and function could have ripple effects across various physiological systems. These findings set the stage for further studies. Longitudinal analyses could explore whether these microbiota differences precede weight changes or result from them. Additionally, mechanistic studies could elucidate how the identified bacterial species influence the BMI z-score and whether modulating their populations can have tangible health benefits.

### 4.3 Difference in analyzing OSA-related gut microbiome with two 16S rRNA sequencing approaches

Our study offers preliminary evidence that full-length and V3–V4 16S rRNA sequencing methods identify significant correlations between certain gut microbiota and polysomnographic parameters in pediatric OSA. Both techniques demonstrated the capability to discern relationships between the composition of gut microbiota and OSA-related parameters. Notably, taxa such as *Bifidobacterium*, *Escherichia*, and *Phocaeicola*, which are either anaerobic or facultative anaerobic and typically reside in the human gastrointestinal tract, were examined. Previous research suggests that anaerobic conditions may promote the proliferation of *Bifidobacterium* and *Escherichia* (80, 81), corroborating our findings of their positive correlations with OSA severity indicators. Conversely, *Phocaeicola*, known for its distinctive oxygen tolerance (82), demonstrates an inverse correlation with the AI, supporting our observations of its relationship with OSA severity. Hence, NGS methods are valuable in elucidating the contribution of specific gut microbes to OSA pathogenesis.

Distinctly, the full-length sequencing provided a more comprehensive insight into potential OSA-associated metabolic pathways compared to the V3–V4 technique. The full-length approach has been instrumental in identifying correlations between microbial metabolism in diverse environments and AI, as well as in discovering and characterizing new microbial lineages that significantly enrich our understanding of microbial diversity (83). The associations of propanoate metabolism and the metabolism of glycine, serine, and threonine with OSA were also observed (84, 85). The impact of peptidoglycan biosynthesis, vital for bacterial structural integrity and stress tolerance (86), in relation to OSA and hypoxic conditions is novel and merits further exploration. These observed differences likely reflect the distinct genomic regions targeted by each sequencing method, indicating that each technique may provide a unique perspective on microbiome complexity. Furthermore, full-length 16S rRNA sequencing could offer a more nuanced understanding of the pathophysiological interplay between OSA and the gut microbiota.

### 4.4 Study limitations

The comparison underscores the importance of method selection in microbiome studies. There are some study limitations. First, the choice of sequencing method and analytic pipeline might lead researchers to different conclusions or emphasize certain findings over others (67, 87). It’s a reminder that while partial-length (such as V3–V4) sequencing is more common and might be more cost-effective, there might be trade-offs in terms of the depth and breadth of data acquired (88). Additionally, expanding the scope to include larger cohorts and other disease-associated microbiomes could enrich the preliminary results presented herein. The observed differences in microbial compositions between the two groups, as revealed by the full-length sequencing, can pave the way for longitudinal studies. Second, although certain bacterial species were more predominant in children with OWO than in children with NW and might play roles in metabolism, appetite regulation, or other physiological processes linked to weight, the cause-effect relationships between gut bacteria and weight categories need further investigations. These studies can assess if the microbial differences are a cause or consequence of being overweight or obese and if modulating the microbiota can have therapeutic benefits. Targeted interventions, such as probiotics or dietary changes, could potentially be designed to modulate the gut microbiota in favor of weight regulation.

### 4.5 Conclusions

The full-length 16S rRNA sequencing offers a promising avenue for microbial research, particularly when high taxonomic resolution is required. However, the choice between it and the V3–V4 region should be context-driven, factoring in the study’s goals, available resources, and the potential trade-offs of each method. Furthermore, the observed advantages of full-length sequencing in differentiating gut microbiota and predictive functions between children with OWO and children with NW, coupled with its ability to correlate these functions with OSA-related indicators, highlight its potential in advancing our understanding of the complex relationship between the gut microbiome and weight. It emphasizes the need for methodological rigor in microbiome meta-analysis and suggests a rich area for future investigations. The associations drawn between gut microbiota composition, metabolic pathways, and BMI z-score offer a promising avenue to understand weight management and overall health better.

## Data availability statement

The original contributions presented in the study are publicly available. This data can be found here: https://doi.org/10.6084/m9.figshare.25959100.

## Ethics statement

The studies involving humans were approved by the Institutional Review Board of Chang Gung Medical Foundation. The studies were conducted in accordance with the local legislation and institutional requirements. Written informed consent for participation in this study was provided by the participants’ legal guardians/next of kin.

## Author contributions

H-HC: Conceptualization, Data curation, Investigation, Methodology, Writing – original draft, Writing – review & editing. C-GH: Conceptualization, Data curation, Investigation, Methodology, Writing – review & editing. S-HC: Data curation, Investigation, Visualization, Writing – original draft. H-YL: Investigation, Supervision, Writing – review & editing. C-CL: Investigation, Writing – original draft. L-AL: Conceptualization, Data curation, Formal analysis, Funding acquisition, Investigation, Project administration, Visualization, Writing – original draft, Writing – review & editing.

## References

[1] Qin J Li R Raes J Arumugam M Burgdorf KS Manichanh C. A human gut microbial gene catalogue established by metagenomic sequencing. Nature. (2010) 464:59–65. doi: 10.1038/nature08821 20203603 PMC3779803

[2] Sangwan N Xia F Gilbert JA. Recovering complete and draft population genomes from metagenome datasets. Microbiome. (2016) 4:8. doi: 10.1186/s40168-016-0154-5 26951112 PMC4782286

[3] Cani PD Van Hul M Lefort C Depommier C Rastelli M Everard A. Microbial regulation of organismal energy homeostasis. Nat Metab. (2019) 1:34–46. doi: 10.1038/s42255-018-0017-4 32694818

[4] Stojanovic O Miguel-Aliaga I Trajkovski M. Intestinal plasticity and metabolism as regulators of organismal energy homeostasis. Nat Metab. (2022) 4:1444–58. doi: 10.1038/s42255-022-00679-6 36396854

[5] Singh RK Chang HW Yan D Lee KM Ucmak D Wong K. Influence of diet on the gut microbiome and implications for human health. J Transl Med. (2017) 15:73. doi: 10.1186/s12967-017-1175-y 28388917 PMC5385025

[6] Chen J Siliceo SL Ni Y Nielsen HB Xu A Panagiotou G. Identification of robust and generalizable biomarkers for microbiome-based stratification in lifestyle interventions. Microbiome. (2023) 11:178. doi: 10.1186/s40168-023-01604-z 37553697 PMC10408196

[7] Popli S Badgujar PC Agarwal T Bhushan B Mishra V. Persistent organic pollutants in foods, their interplay with gut microbiota and resultant toxicity. Sci Total Environ. (2022) 832:155084. doi: 10.1016/j.scitotenv.2022.155084 35395291

[8] Young VB. The role of the microbiome in human health and disease: an introduction for clinicians. BMJ. (2017) 356:j831. doi: 10.1136/bmj.j831 28298355

[9] Stanislawski MA Dabelea D Lange LA Wagner BD Lozupone CA. Gut microbiota phenotypes of obesity. NPJ Biofilms Microbiomes. (2019) 5:18. doi: 10.1038/s41522-019-0091-8 31285833 PMC6603011

[10] Indiani C Rizzardi KF Castelo PM Ferraz LFC Darrieux M Parisotto TM. Childhood obesity and firmicutes/bacteroidetes ratio in the gut microbiota: a systematic review. Child Obes. (2018) 14:501–9. doi: 10.1089/chi.2018.0040 30183336

[11] Gong J Shen Y Zhang H Cao M Guo M He J. Gut microbiota characteristics of people with obesity by meta-analysis of existing datasets. Nutrients. (2022) 14:2993. doi: 10.3390/nu14142993 35889949 PMC9325184

[12] Magne F Gotteland M Gauthier L Zazueta A Pesoa S Navarrete P. The firmicutes/bacteroidetes ratio: a relevant marker of gut dysbiosis in obese patients? Nutrients. (2020) 12:1474. doi: 10.3390/nu12051474 32438689 PMC7285218

[13] Heiss CN Olofsson LE. Gut microbiota-dependent modulation of energy metabolism. J Innate Immun. (2018) 10:163–71. doi: 10.1159/000481519 PMC6757175 29131106

[14] Ridlon JM Kang DJ Hylemon PB. Bile salt biotransformations by human intestinal bacteria. J Lipid Res. (2006) 47:241–59. doi: 10.1194/jlr.R500013-JLR200 16299351

[15] Cheng Z Zhang L Yang L Chu H. The critical role of gut microbiota in obesity. Front Endocrinol (Lausanne). (2022) 13:1025706. doi: 10.3389/fendo.2022.1025706 36339448 PMC9630587

[16] Nemergut DR Costello EK Hamady M Lozupone C Jiang L Schmidt SK. Global patterns in the biogeography of bacterial taxa. Environ Microbiol. (2011) 13:135–44. doi: 10.1111/j.1462-2920.2010.02315.x PMC5834236 21199253

[17] Janda JM Abbott SL. 16S rRNA gene sequencing for bacterial identification in the diagnostic laboratory: pluses, perils, and pitfalls. J Clin Microbiol. (2007) 45:2761–4. doi: 10.1128/JCM.01228-07 PMC2045242 17626177

[18] Ramazzotti M Bacci G. Chapter 5 - 16S rRNA-Based Taxonomy Profiling in the Metagenomics Era. In: Nagarajan M editor. Metagenomics. Academic Press (2018), 103–19. doi: 10.1016/B978-0-08-102268-9.00005-7

[19] Hiergeist A Ruelle J Emler S Gessner A. Reliability of species detection in 16S microbiome analysis: Comparison of five widely used pipelines and recommendations for a more standardized approach. PloS One. (2023) 18:e0280870. doi: 10.1371/journal.pone.0280870 36795699 PMC9934417

[20] Myer PR McDaneld TG Kuehn LA Dedonder KD Apley MD Capik SF. Classification of 16S rRNA reads is improved using a niche-specific database constructed by near-full length sequencing. PloS One. (2020) 15:e0235498. doi: 10.1371/journal.pone.0235498 32658916 PMC7357769

[21] Zhang K Lin R Chang Y Zhou Q Zhang Z. 16S-FASAS: an integrated pipeline for synthetic full-length 16S rRNA gene sequencing data analysis. PeerJ. (2022) 10:e14043. doi: 10.7717/peerj.14043 36172503 PMC9511998

[22] Katiraei S Anvar Y Hoving L Berbee JFP van Harmelen V Willems van Dijk K. Evaluation of full-length versus V4-region 16S rRNA sequencing for phylogenetic analysis of mouse intestinal microbiota after a dietary intervention. Curr Microbiol. (2022) 79:276. doi: 10.1007/s00284-022-02956-9 35907023 PMC9338901

[23] Mukherjee C Beall CJ Griffen AL Leys EJ. High-resolution ISR amplicon sequencing reveals personalized oral microbiome. Microbiome. (2018) 6:153. doi: 10.1186/s40168-018-0535-z 30185233 PMC6126016

[24] Lopez-Aladid R Fernandez-Barat L Alcaraz-Serrano V Bueno-Freire L Vazquez N Pastor-Ibanez R. Determining the most accurate 16S rRNA hypervariable region for taxonomic identification from respiratory samples. Sci Rep. (2023) 13:3974. doi: 10.1038/s41598-023-30764-z 36894603 PMC9998635

[25] Bertolo A Valido E Stoyanov J. Optimized bacterial community characterization through full-length 16S rRNA gene sequencing utilizing MinION nanopore technology. BMC Microbiol. (2024) 24:58. doi: 10.1186/s12866-024-03208-5 38365589 PMC10870487

[26] Pootakham W Mhuantong W Yoocha T Sangsrakru D Kongkachana W Sonthirod C. Taxonomic profiling of Symbiodiniaceae and bacterial communities associated with Indo-Pacific corals in the Gulf of Thailand using PacBio sequencing of full-length ITS and 16S rRNA genes. Genomics. (2021) 113:2717–29. doi: 10.1016/j.ygeno.2021.06.001 34089786

[27] Jeong J Yun K Mun S Chung WH Choi SY Nam YD. The effect of taxonomic classification by full-length 16S rRNA sequencing with a synthetic long-read technology. Sci Rep. (2021) 11:1727. doi: 10.1038/s41598-020-80826-9 33462291 PMC7814050

[28] Dong S Jiao J Jia S Li G Zhang W Yang K. 16S rDNA full-length assembly sequencing technology analysis of intestinal microbiome in polycystic ovary syndrome. Front Cell Infect Microbiol. (2021) 11:634981. doi: 10.3389/fcimb.2021.634981 34041041 PMC8141595

[29] Karst SM Dueholm MS McIlroy SJ Kirkegaard RH Nielsen PH Albertsen M. Retrieval of a million high-quality, full-length microbial 16S and 18S rRNA gene sequences without primer bias. Nat Biotechnol. (2018) 36:190–5. doi: 10.1038/nbt.4045 29291348

[30] Lo Bue A Salvaggio A Insalaco G. Obstructive sleep apnea in developmental age. A narrative review Eur J Pediatr. (2020) 179:357–65. doi: 10.1007/s00431-019-03557-8 31940071

[31] Chuang HH Hsu JF Chuang LP Chen NH Huang YS Li HY. Differences in anthropometric and clinical features among preschoolers, school-age children, and adolescents with obstructive sleep apnea-A hospital-based study in Taiwan. Int J Environ Res Public Health. (2020) 17:4663. doi: 10.3390/ijerph17134663 32610444 PMC7370095

[32] Yan W Jiang M Hu W Zhan X Liu Y Zhou J. Causality investigation between gut microbiota, derived metabolites, and obstructive sleep apnea: A bidirectional mendelian randomization study. Nutrients. (2023) 15:4544. doi: 10.3390/nu15214544 37960197 PMC10648878

[33] Chuang HH Hsu JF Chuang LP Chiu CH Huang YL Li HY. Different associations between tonsil microbiome, chronic tonsillitis, and intermittent hypoxemia among obstructive sleep apnea children of different weight status: A pilot case-control Study. J Pers Med. (2021) 11:486. doi: 10.3390/jpm11060486 34071547 PMC8227284

[34] World Medical A. World Medical Association Declaration of Helsinki: ethical principles for medical research involving human subjects. JAMA. (2013) 310:2191–4. doi: 10.1001/jama.2013.281053 24141714

[35] von Elm E Altman DG Egger M Pocock SJ Gotzsche PC Vandenbroucke JP. The Strengthening the Reporting of Observational Studies in Epidemiology (STROBE) statement: guidelines for reporting observational studies. PloS Med. (2007) 4:e296. doi: 10.1371/journal.pmed.0040296 17941714 PMC2020495

[36] Kaditis A Kheirandish-Gozal L Gozal D. Algorithm for the diagnosis and treatment of pediatric OSA: a proposal of two pediatric sleep centers. Sleep Med. (2012) 13:217–27. doi: 10.1016/j.sleep.2011.09.009 22300748

[37] Chuang HH Huang CG Chuang LP Huang YS Chen NH Li HY. Relationships among and predictive values of obesity, inflammation markers, and disease severity in pediatric patients with obstructive sleepapnea before and after adenotonsillectomy. J Clin Med. (2020) 9:579. doi: 10.3390/jcm9020579 32093397 PMC7073666

[38] de Onis M Onyango AW Borghi E Siyam A Nishida C Siekmann J. Development of a WHO growth reference for school-aged children and adolescents. Bull World Health Organization. (2007) 85:660–7. doi: 10.2471/blt.07.043497 PMC2636412 18026621

[39] Berry RB Budhiraja R Gottlieb DJ Gozal D Iber C Kapur VK. Rules for scoring respiratory events in sleep: update of the 2007 AASM Manual for the Scoring of Sleep and Associated Events. Deliberations of the Sleep Apnea Definitions Task Force of the American Academy of Sleep Medicine. J Clin Sleep Med. (2012) 8:597–619. doi: 10.5664/jcsm.2172 23066376 PMC3459210

[40] Lee LA Chuang HH Hsieh HS Wang CY Chuang LP Li HY. Using sleep heart rate variability to investigate the sleep quality in children with obstructive sleep apnea. Front Public Health. (2023) 11:1103085. doi: 10.3389/fpubh.2023.1103085 36923030 PMC10008856

[41] Huang CG Hsu JF Chuang LP Li HY Fang TJ Huang YS. Adenotonsillectomy-related changes in systemic inflammation among children with obstructive sleep apnea. J Chin Med Assoc. (2023) 86:596–605. doi: 10.1097/JCMA.0000000000000921 36989493 PMC12755525

[42] PacBio. Amplification of bacterial full-length 16S gene with barcoded primers (2022). Available at: https://www.pacb.com/wp-content/uploads/Procedure-Checklist-%E2%80%93-Amplification-of-Full-Length-16S-Gene-with-Barcoded-Primers-for-Multiplexed-SMRTbell-Library-Preparation-and-Sequencing.pdf.

[43] Wagner J Coupland P Browne HP Lawley TD Francis SC Parkhill J. Evaluation of PacBio sequencing for full-length bacterial 16S rRNA gene classification. BMC Microbiol. (2016) 16:274. doi: 10.1186/s12866-016-0891-4 27842515 PMC5109829

[44] Callahan BJ McMurdie PJ Rosen MJ Han AW Johnson AJ Holmes SP. DADA2: High-resolution sample inference from Illumina amplicon data. Nat Methods. (2016) 13:581–3. doi: 10.1038/nmeth.3869 PMC4927377 27214047

[45] Bokulich NA Kaehler BD Rideout JR Dillon M Bolyen E Knight R. Optimizing taxonomic classification of marker-gene amplicon sequences with QIIME 2’s q2-feature-classifier plugin. Microbiome. (2018) 6:90. doi: 10.1186/s40168-018-0470-z 29773078 PMC5956843

[46] Rognes T Flouri T Nichols B Quince C Mahe F. VSEARCH: a versatile open source tool for metagenomics. PeerJ. (2016) 4:e2584. doi: 10.7717/peerj.2584 27781170 PMC5075697

[47] Bolyen E Rideout JR Dillon MR Bokulich NA Abnet CC Al-Ghalith GA. Reproducible, interactive, scalable and extensible microbiome data science using QIIME 2. Nat Biotechnol. (2019) 37:852–7. doi: 10.1038/s41587-019-0209-9 PMC7015180 31341288

[48] Katoh K Standley DM. MAFFT multiple sequence alignment software version 7: improvements in performance and usability. Mol Biol Evol. (2013) 30:772–80. doi: 10.1093/molbev/mst010 PMC3603318 23329690

[49] Price MN Dehal PS Arkin AP. FastTree: computing large minimum evolution trees with profiles instead of a distance matrix. Mol Biol Evol. (2009) 26:1641–50. doi: 10.1093/molbev/msp077 PMC2693737 19377059

[50] Martin M. Cutadapt removes adapter sequences from high-throughput sequencing reads. EMBnetjournal. (2011) 17:10–2. doi: 10.14806/ej.17.1.200

[51] Lozupone CA Knight R. Species divergence and the measurement of microbial diversity. FEMS Microbiol Rev. (2008) 32:557–78. doi: 10.1111/j.1574-6976.2008.00111.x PMC2443784 18435746

[52] Lozupone C Lladser ME Knights D Stombaugh J Knight R. UniFrac: an effective distance metric for microbial community comparison. ISME J. (2011) 5:169–72. doi: 10.1038/ismej.2010.133 PMC3105689 20827291

[53] Jiang XT Peng X Deng GH Sheng HF Wang Y Zhou HW. Illumina sequencing of 16S rRNA tag revealed spatial variations of bacterial communities in a mangrove wetland. Microb Ecol. (2013) 66:96–104. doi: 10.1007/s00248-013-0238-8 23649297

[54] Noval Rivas M Burton OT Wise P Zhang YQ Hobson SA Garcia Lloret M. A microbiota signature associated with experimental food allergy promotes allergic sensitization and anaphylaxis. J Allergy Clin Immunol. (2013) 131:201–12. doi: 10.1016/j.jaci.2012.10.026 PMC3860814 23201093

[55] Parks DH Tyson GW Hugenholtz P Beiko RG. STAMP: statistical analysis of taxonomic and functional profiles. Bioinformatics. (2014) 30:3123–4. doi: 10.1093/bioinformatics/btu494 PMC4609014 25061070

[56] Segata N Izard J Waldron L Gevers D Miropolsky L Garrett WS. Metagenomic biomarker discovery and explanation. Genome Biol. (2011) 12:R60. doi: 10.1186/gb-2011-12-6-r60 21702898 PMC3218848

[57] Schmidt M Unterer S Suchodolski JS Honneffer JB Guard BC Lidbury JA. The fecal microbiome and metabolome differs between dogs fed Bones and Raw Food (BARF) diets and dogs fed commercial diets. PloS One. (2018) 13:e0201279. doi: 10.1371/journal.pone.0201279 30110340 PMC6093636

[58] Zhu Z Satten GA Mitchell C Hu YJ. Constraining PERMANOVA and LDM to within-set comparisons by projection improves the efficiency of analyses of matched sets of microbiome data. Microbiome. (2021) 9:133. doi: 10.1186/s40168-021-01034-9 34108046 PMC8191060

[59] Asshauer KP Wemheuer B Daniel R Meinicke P. Tax4Fun: predicting functional profiles from metagenomic 16S rRNA data. Bioinformatics. (2015) 31:2882–4. doi: 10.1093/bioinformatics/btv287 PMC4547618 25957349

[60] Kanehisa M Furumichi M Tanabe M Sato Y Morishima K. KEGG: new perspectives on genomes, pathways, diseases and drugs. Nucleic Acids Res. (2017) 45:D353–61. doi: 10.1093/nar/gkw1092 PMC5210567 27899662

[61] Liu C Cui Y Li X Yao M. microeco: an R package for data mining in microbial community ecology. FEMS Microbiol Ecol. (2021) 97:fiaa255. doi: 10.1093/femsec/fiaa255 33332530

[62] Matsuo Y Komiya S Yasumizu Y Yasuoka Y Mizushima K Takagi T. Full-length 16S rRNA gene amplicon analysis of human gut microbiota using MinION nanopore sequencing confers species-level resolution. BMC Microbiol. (2021) 21:35. doi: 10.1186/s12866-021-02094-5 33499799 PMC7836573

[63] Johnson JS Spakowicz DJ Hong BY Petersen LM Demkowicz P Chen L. Evaluation of 16S rRNA gene sequencing for species and strain-level microbiome analysis. Nat Commun. (2019) 10:5029. doi: 10.1038/s41467-019-13036-1 31695033 PMC6834636

[64] Chiarello M McCauley M Villeger S Jackson CR. Ranking the biases: The choice of OTUs vs. ASVs in 16S rRNA amplicon data analysis has stronger effects on diversity measures than rarefaction and OTU identity threshold. PloS One. (2022) 17:e0264443. doi: 10.1371/journal.pone.0264443 35202411 PMC8870492

[65] Cameron ES Schmidt PJ Tremblay BJ Emelko MB Muller KM. Enhancing diversity analysis by repeatedly rarefying next generation sequencing data describing microbial communities. Sci Rep. (2021) 11:22302. doi: 10.1038/s41598-021-01636-1 34785722 PMC8595385

[66] Callahan BJ Grinevich D Thakur S Balamotis MA Yehezkel TB. Ultra-accurate microbial amplicon sequencing with synthetic long reads. Microbiome. (2021) 9:130. doi: 10.1186/s40168-021-01072-3 34090540 PMC8179091

[67] Nearing JT Douglas GM Hayes MG MacDonald J Desai DK Allward N. Microbiome differential abundance methods produce different results across 38 datasets. Nat Commun. (2022) 13:342. doi: 10.1038/s41467-022-28034-z 35039521 PMC8763921

[68] Neu AT Allen EE Roy K. Defining and quantifying the core microbiome: Challenges and prospects. Proc Natl Acad Sci USA. (2021) 118:e2104429118. doi: 10.1073/pnas.2104429118 34862327 PMC8713806

[69] Adeolu M Parkinson J Xiong X. Analyzing metabolic pathways in microbiomes. Methods Mol Biol. (2018) 1849:291–307. doi: 10.1007/978-1-4939-8728-3_18 30298261

[70] Vijay A Valdes AM. Role of the gut microbiome in chronic diseases: a narrative review. Eur J Clin Nutr. (2022) 76:489–501. doi: 10.1038/s41430-021-00991-6 34584224 PMC8477631

[71] de Vos WM Tilg H Van Hul M Cani PD. Gut microbiome and health: mechanistic insights. Gut. (2022) 71:1020–32. doi: 10.1136/gutjnl-2021-326789 PMC8995832 35105664

[72] Hosomi K Saito M Park J Murakami H Shibata N Ando M. Oral administration of Blautia wexlerae ameliorates obesity and type 2 diabetes via metabolic remodeling of the gut microbiota. Nat Commun. (2022) 13:4477. doi: 10.1038/s41467-022-32015-7 35982037 PMC9388534

[73] Hill EB Konigsberg IR Ir D Frank DN Jambal P Litkowski EM. The microbiome, epigenome, and diet in adults with obesity during behavioral weight loss. Nutrients. (2023) 15:3588. doi: 10.3390/nu15163588 37630778 PMC10458964

[74] Li L Wang Y Yuan J Liu Z Ye C Qin S. Undaria pinnatifida improves obesity-related outcomes in association with gut microbiota and metabolomics modulation in high-fat diet-fed mice. Appl Microbiol Biotechnol. (2020) 104:10217–31. doi: 10.1007/s00253-020-10954-9 33074417

[75] Cano PG Santacruz A Trejo FM Sanz Y. Bifidobacterium CECT 7765 improves metabolic and immunological alterations associated with obesity in high-fat diet-fed mice. Obes (Silver Spring). (2013) 21:2310–21. doi: 10.1002/oby.20330 23418126

[76] Gonzalez-Vazquez R Zuniga-Leon E Torres-Maravilla E Leyte-Lugo M Mendoza-Perez F Hernandez-Delgado NC. Genomic and biochemical characterization of bifidobacterium pseudocatenulatum JCLA3 isolated from human intestine. Microorganisms. (2022) 10:2100. doi: 10.3390/microorganisms10112100 36363691 PMC9695335

[77] Whiley RA Fraser HY Douglas CW Hardie JM Williams AM Collins MD. Streptococcus parasanguis sp. nov., an atypical viridans Streptococcus from human clinical specimens. FEMS Microbiol Lett. (1990) 56:115–21. doi: 10.1111/j.1574-6968.1990.tb04133.x 1692001

[78] Zheng D Liwinski T Elinav E. Interaction between microbiota and immunity in health and disease. Cell Res. (2020) 30:492–506. doi: 10.1038/s41422-020-0332-7 32433595 PMC7264227

[79] Shoubridge AP Choo JM Martin AM Keating DJ Wong ML Licinio J. The gut microbiome and mental health: advances in research and emerging priorities. Mol Psychiatry. (2022) 27:1908–19. doi: 10.1038/s41380-022-01479-w 35236957

[80] Shimamura S Abe F Ishibashi N Miyakawa H Yaeshima T Araya T. Relationship between oxygen sensitivity and oxygen metabolism of Bifidobacterium species. J Dairy Sci. (1992) 75:3296–306. doi: 10.3168/jds.S0022-0302(92)78105-3 1474198

[81] McDaniel LE Bailey EG Zimmerli A. Effect of oxygen supply rates on growth of escherichia coli. Appl Microbiol. (1965) 13:109–14. doi: 10.1128/am.13.1.109-114.1965 PMC1058202 14264837

[82] Keitel L Braun K Finger M Kosfeld U Yordanov S Buchs J. Carbon dioxide and trace oxygen concentrations impact growth and product formation of the gut bacterium Phocaeicola vulgatus. BMC Microbiol. (2023) 23:391. doi: 10.1186/s12866-023-03127-x 38062358 PMC10701953

[83] Shu WS Huang LN. Microbial diversity in extreme environments. Nat Rev Microbiol. (2022) 20:219–35. doi: 10.1038/s41579-021-00648-y 34754082

[84] Abbes S Baldi S Sellami H Amedei A Keskes L. Molecular methods for colorectal cancer screening: Progress with next-generation sequencing evolution. World J Gastrointest Oncol. (2023) 15:425–42. doi: 10.4251/wjgo.v15.i3.425 PMC10052664 37009313

[85] Humer E Pieh C Brandmayr G. Metabolomics in sleep, insomnia and sleep apnea. Int J Mol Sci. (2020) 21:7244. doi: 10.3390/ijms21197244 33008070 PMC7583860

[86] Shaku M Ealand C Matlhabe O Lala R Kana BD. Peptidoglycan biosynthesis and remodeling revisited. Adv Appl Microbiol. (2020) 112:67–103. doi: 10.1016/bs.aambs.2020.04.001 32762868

[87] Varadi L Luo JL Hibbs DE Perry JD Anderson RJ Orenga S. Methods for the detection and identification of pathogenic bacteria: past, present, and future. Chem Soc Rev. (2017) 46:4818–32. doi: 10.1039/C6CS00693K 28644499

[88] Abellan-Schneyder I MatChado MS Reitmeier S Sommer A Sewald Z Baumbach J. Primer, pipelines, parameters: issues in 16S rRNA gene sequencing. mSphere. (2021) 6:e01202–20. doi: 10.1128/mSphere.01202-20 PMC8544895 33627512

